# Role of sperm apoptosis and oxidative stress in male infertility: A narrative review

**DOI:** 10.18502/ijrm.v19i6.9371

**Published:** 2021-07-27

**Authors:** Asadollah Asadi, Rozita Ghahremani, Arash Abdolmaleki, Farzad Rajaei

**Affiliations:** ^1^Department of Biology, Faculty of Science, University of Mohaghegh Ardabili, Ardabil, Iran.; ^2^Department of Engineering Sciences, Faculty of Advanced Technologies, University of Mohaghegh Ardabili, Namin, Iran.; ^3^BioScience and Biotechnology Research Center (BBRC), Sabalan University of Advanced Technologies (SUAT), Namin, Iran.; ^4^Cellular and Molecular Research Center, Qazvin University of Medical Sciences, Qazvin, Iran.

**Keywords:** Fertility, Spermatogonia, Apoptosis, Reproduction, DNA fragmentation, DNA integrity, ROS.

## Abstract

Activation of caspase, externalization of phosphatidyl serine, change in the mitochondrial membrane potential, and DNA fragmentation are apoptosis markers found in human ejaculated spermatozoa. Also, reactive oxygen species (ROS) play a vital role in the different types of male infertility. In this review, data sources including Google Scholar, Scopus, PubMed, and Science Direct were searched for publications with no particular time restriction to get a holistic and comprehensive view of the research. Apoptosis regulates the male germ cells, correct function and development from the early embryonic stages of gonadal differentiation to fertilization. In addition to maintaining a reasonable ratio between the Sertoli and germ cells, apoptosis is one of the well-known quality control mechanisms in the testis. Also, high ROS levels cause a heightened and dysregulated apoptotic response. Apoptosis is one of the well-known mechanisms of quality control in the testis. Nevertheless, increased apoptosis may have adverse effects on sperm production. Recent studies have shown that ROS and the consequent oxidative stress play a crucial role in apoptosis. This review aims to assimilate and summarize recent findings on the apoptosis in male reproduction and fertility. Also, this review discusses the update on the role of ROS in normal sperm function to guide future research in this area.

## 1. Introduction

Infertility is an important medical and behavioral issue that affects many people. Apoptosis is an important physiological mechanism which has been shown to play significant roles in various physiological processes. Apoptosis, which is characterized by chromatin disintegration during programmed cell death, is a distinct mechanism that leads to DNA fragmentation. This biological process is critical for proper germ cell formation and the maintenance of the germ cell-Sertoli ratio in the testis (1, 2). In the 1940s, reactive oxygen species (ROS) were reported as a potential contributor to male infertility (3). Oxidative stress (OS) leads to defect sperm operation. Recent studies have shown that ROS can be a contribute factor in 30-80% of infertile males (4). OS is caused by an imbalance in the physiology of body between antioxidants and ROS (5). The major causes of DNA damage are aberrant apoptosis and OS. DNA fragmentation is a common result of ROS-mediated damage, and it's most commonly seen in infertile men's spermatozoa. Direct or indirect ROS-mediated damage can result in single or double-stranded fragments and abnormal apoptosis (1). Semen analysis is an important first step in the laboratory assessment of an infertile male (6). It includes evaluation of the volume of ejaculates and sperm quantity, motility, and shape by the World Health Organization criteria (7). The effects of male elderly on quality of semen, DNA breakage, and chromosomal abnormalities have been studied since 1970 have been reported in infertile patients and fertile donors but the results are conflicting (8). Pollutants in nature cause harmful effects on sperm motility, vitality, membrane lipid composition, and acrosome status and is related to excessive ROS production (9).

This review aimed to assimilate and summarize recent findings of apoptosis as an important physiological process in male infertility.

## 2. Materials and Methods

### Search strategy

This study is a narrative review and the data were retrieved from Google Scholar, PubMed, Scopus and Science Direct. Publications were searched with no particular time restriction from 1943 to 2020 to get a holistic and comprehensive view of the research done on this topic so far with the following terms: “Apoptosis", “Fertility", “Male infertility", “Mitochondria in the apoptosis", “Spermatogenesis", “Apoptosis in a male germ cell", “Apoptosis pathways", “Hormones, and germ cell apoptosis", “Sperm morphology and ROS production", “Sperm apoptosis", “Sperm DNA integrity", and “DNA fragmentation Apoptosis, and semen quality".

### Study selection

The study was done in three steps: first, the titles of the papers were searched according to the selected terms, and appropriate titles were selected for the next step. Second, abstracts were reviewed and eligible papers were selected. At the last step, full-texts of the eligible papers were evaluated. In total, 1,253 papers were evaluated, of which, 1,168 were excluded because of no consistency with the study goals or no new important data. Finally, 85 papers were included in this review. Also, 30 papers were omitted because of the language of the papers (such as Turkish or Arabic).

## 3. Results

### Spermatogenesis

Spermatogenesis is one of the most active self-renewal processes in the body: 10${}^{7}$ sperm per day are produced per gram of testicular tissue. The time taken to complete the cycle is unique and unalterable for any mammalian species. Since the process is supported by somatic Sertoli cells, cell-cell interaction between the germ and Sertoli cells is generally thought to control the duration of cell cycles and cell organization (10-12). Spermatogenesis is divided into three stages: (i): spermatogonia duplication and distinction; (ii) meiosis, and (iii) spermiogenesis, a complex mechanism which transforms round spermatids after meiosis into a complex structure called the spermatozoon. In humans, the spermatogenesis process begins at the time of puberty and continues throughout a men's lifetime (13). During this process, an early wave of apoptosis that follows the first round of spermatogenesis in the testes will eliminate excess spermatogonia (14). Disorder in apoptosis results in a phenotype of male infertility due to an imbalance in germ- and Sertoli cell numbers (15). Later during the life, apoptosis plays a role in the elimination of germ cells that are damaged by exposure to environmental toxicants, chemotherapy drugs, or heat (16). Almost 75% of the spermatogonia die in the process of apoptosis before maturity (17, 18). Therefore, apoptosis plays a significant role in controlling the spermatogenesis of different species of mammals, including humans (19). High apoptosis levels have been reported in infertile men testicular biopsies (20). Although spermatogenesis is affected by the hypothalamic-pituitary-testicular axis, exogenous factors such as infections, exposure to heavy metals, smoking of cigarettes, irradiation, chemicals/herbs, and medicines impair spermatogenesis and predispose sperm cells to harm.

### Apoptosis

Apoptosis is a programmed cell death that involves the removal of genetically damaged cells (2). In the lack of special cell surface receptors, factors that can penetrate the cell straight and modulate the apoptotic cascade may cause apoptotic activation (21). Such factors include: heat shock, stressors, ROS, ultraviolet radiation, drug, synthetic peptides, and toxins (22). Nowadays, the attendance and activity of apoptotic signals in human sperm in response to different stimuli are widely accepted (23, 24). There are two distinct mechanisms for apoptosis initiation: extrinsic pathway or receptor apoptosis and apoptosis endogenous or mitochondrial (25). Specific mechanisms include the perforin-granzyme A and B pathway and P53 pathway that induces apoptosis (26, 27). The biochemical particularity of apoptosis consists of the transmission of phosphatidylserine to the plasma membrane external, caspase activation, and DNA fragmentation (28). Caspase activity is associated with sperm immaturity, low numbers, decreased motilities (29, 30), lower fertilization levels (31), and lack of plasma membrane integrity, as demonstrated by externalization of phosphatidylserine (32). Caspases are cysteine proteases which promote apoptosis in mammals (33). Apoptosis cycle is significant in the background of germ cells because both mitosis and meiosis occur in cells, and cell death may be necessary to remove cells with genetic defects during the process (34). The proportion of apoptotic sperm in infertile men's ejaculated semen samples is reported to be higher than in healthy men (35). Furthermore, during cryopreservation, infertile patients' sperm caspases become more active than healthy donors' (36). However, it is unclear whether the apoptotic markers detected in spermatozoa are remnants of an unsuccessful apoptotic cycle that occurred before to ejaculation, or if they are the product of apoptosis that occurred after ejaculation (37).

### The role of mitochondria in the apoptosis process

Mitochondria are organelles that participate in the ATP synthesis, calcium signals, production of ROS, and regulation of apoptosis (21). Mitochondrial abnormalities trigger physiological disorders, including infertility (38). The mitochondria function at the heart of the apoptotic pathway by offering main factors, including those which trigger caspase activity and DNA fragmentation (39). Caspases are a family of proteases that are essential for the regulation of apoptosis (40). Cytochrome c, which is one of the main factors of apoptosis, mediates caspase 9 and caspase 3 activations, which leads to cell suicide (41). *Bcl-2* is a family of regularizer proteins that play a role in the control of mitochondrial permeability and, therefore, in apoptosis regulation (2).

### Apoptosis in male germ cell 

#### Internal pathway of apoptosis

Primordial germ cells are sourced from the epiblast and finally migrate to the gonad. Surplus cells produced during this time are killed by apoptosis, which depends mainly on the harmony between *Bcl-xL* and *Bax* (42). The early wave of apoptosis removed in transgenic mice with overexpressing *Bcl-2* or *Bclx* results in the cumulation of spermatogonia and spermatocytes, further resulting in infertile animals (43). *Bcl-x*-knockout mice had severe defects in male germ cells during growth (44). Therefore, adult rats with two mutant *Bcl-x* alleles lack spermatogonia (42). *Bcl-w* mutant mice show almost perfect degeneration of the testicles (34). Although *Bax*, *Bcl-w*, and *Bcl-2* are the primary regulators of germ cell growth and maturity post-birth, *Bcl-x* is necessary for the durability of primordial germ cells in the embryonic gonad during early phases (45).

#### External pathway of apoptosis

*Fas* is a transmembrane molecule with 281 amino acids which are activated by *FasL* and intercede apoptosis. *FasL* and respective receptor *Fas* both interact, and the activated *Fas* induce apoptosis in the cell (46). It is widely accepted that Sertoli cells regulate the number of germ cells by one of the most common apoptotic pathways, the *Fas*/*FasL* paracrine signal transmission machine, in which the *FasL* expressed in Sertoli cells and Fas expressed in germ cells induce apoptosis when connecting with each other (47). Mice with a random loss of function mutation in the *Fas* gene (Fas${}^{\mathrm{lpr}}$) or *FasL* (Fasl${}^{\mathrm{gld}}$) develop apparent lymphoproliferative and autoimmune diseases. The consequence of the *Fas/FasL*-mediated cycle is hypospermatogenesis, such as maturity arrest and Sertoli cell syndrome. Germ cell maturity may be associated with *Fas* gene expression that is able to induce apoptosis to eliminate damaged germ cells (48). The expression of *Fas*/*FasL* in the human testis is regulated by gonadotropin. It is generally proven that the *Fas*/*FasL* system may have a role in the quality-control process of the manufactured gametes (34).

### Hormones and germ cell apoptosis

**Figure 1:**
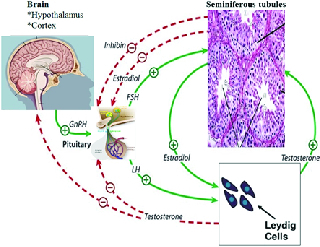
Effects of hormones on the germ cells survival. LH helps with steroid genesis by activating Leydig cells but FSH activates the Sertoli cells to help the developmental stages of spermatogenesis.

In the mammalian testes, follicle-stimulating hormone (FSH), luteinizing hormone, gonadotropin, and testosterone regulate germ cells proliferation, differentiation, and viability (34). Luteinizing hormone helps with steroidogenesis by activating Leydig cells, but FSH activates the Sertoli cells to help with the spermatogenesis developmental stages (13). Getting exposed to excess hormones or hormone deficiency may result in cellular apoptosis in the testis (49). Sertoli cells have FSH and testosterone receptors, which are the major spermatogenesis hormonal regulators. Removal of the hormone causes apoptosis of germ cells (50). While testosterone and the synergistic activity of FSH with estradiol help germ cell survival during seminiferous tubular maturity, estradiol alone has an inhibitory effect and is an inducer of apoptosis (Figure 1) (34). Besides, excess testosterone can lead to increased expression of *Fas*/*FasL* in testis (51). Testosterone removal stimulates caspase activity and results in DNA fragmentation in Sertoli cells (52).

### Sperm apoptosis

In the seminiferous epithelium, spermatogenesis is accompanied by germ cell apoptosis, a cycle that generally takes place during life. Apoptosis of germ cells is essential to retain the optimal germ cell ratio to Sertoli cells and to remove abnormal germ cells, especially during maturity. The exit of phosphatidylserine to the outer membrane of the sperm, caspases activation, and chromosome fragmentation are considered to mark apoptosis (53). *Bcl-2*, the apoptosis inhibitor gene, safeguards the cell by reducing the ROS generation. Although the *FAS* receptor frequently causes apoptosis, this fails to clean all the sperm intended for elimination, leading to a high population of defective sperm. The percentage of *FAS*-positive sperm can be > 50% in men with unusual sperm parameters (5).

### Role of apoptosis in male infertility

In certain pathological circumstances, an enormous increase in germ cell apoptosis occurs, which involves idiopathic infertility in males (54). Apoptosis has been observed often in spermatocytes, less in spermatogonia, and rarely in spermatids (55). Fujisawa and co-workers documented the existence of apoptosis in the testes, especially in spermatocytes (56). A study by Martincic and colleagues determined the presence and abundance of germ cells apoptosis in infertile males whereas Sertoli cells do not undergo apoptosis (18). It has been shown that the *Fas*-system is involved in regulating the spontaneous apoptosis of germ cells. In the normal state, Sertoli cells express *FasL* which triggers the apoptosis in *Fas*-positive germ cells, and shows a paracrine interaction between germ and Sertoli cells (57, 58). Apoptosis increases with age in the testes, resulting in a decrease in germ cells. This may be linked with the reduction in androgen levels or a rise in OS (21). Measuring apoptosis rates can also be used as a sign for pursuing male infertility treatment (59).

### The role of OS in apoptosis

OS is one of the major causes of male infertility. In recent years, the production of ROS and its influence on semen quality have been widely studied. OS occurs as a result of an imbalance between ROS production and antioxidants (1, 60).

Mitochondrial exposure to ROS leads to the induction of apoptosis and thus causes the fragmentation of the DNA (19, 61). A direct association between increased sperm damage caused by ROS and high levels of cytochrome C, caspase 9, and 3 was demonstrated in the study by Soderquist and colleagues which showed positive apoptosis in infertile men. Studies in infertile men have shown that high levels of cytochrome C in plasma show remarkable mitochondrial harm by ROS (62). Increased age causes ROS to accumulate which induces lipid peroxidation. Excessive amounts of ROS and reduced antioxidant capacity during ageing can cause apoptosis or oxidative damage to DNA (63). The damaged paternal DNA, if not repaired, may through fertilization reach the couple's offspring, causing a variety of diseases (64). In this regard, certain clinical research have found that giving antioxidants improves sperm DNA integrity. Vitamin C, vitamin E, and glutathione, when taken together for two months, dramatically reduced the levels of 8OHdG, a marker for OS-induced sperm DNA damage (65).

### Effect of sperm morphology on ROS production

**Figure 2:**
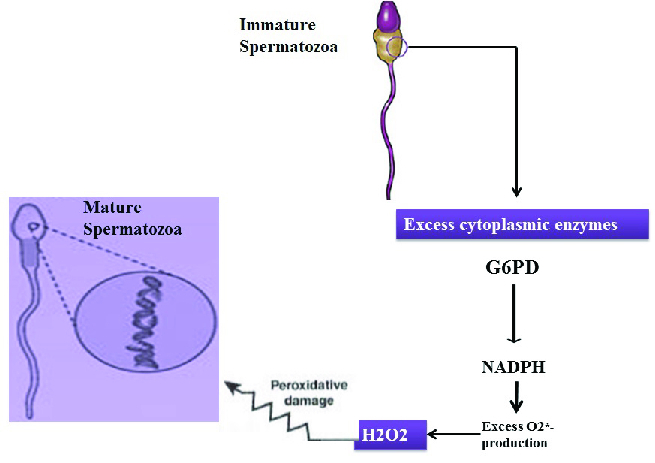
Effects of sperm morphology on the production of endogenous ROS. Figure shows the mechanism for the link between OS and sperm DNA damage. OS leads to damage of the sperm DNA.

Teratozoospermia is caused by a defect in the spermatogenesis process and is determined by sperm with an excess cytoplasm (65). Remaining cytoplasm stimulates sperms to produce endogenous ROS through processes that can be mediated by the enzyme glucose-6-phosphate dehydrogenase. Teratozoospermia patients are at higher risk of pathogenic ROS, apoptosis, and DNA damage (Figure 2) (66). ROS generation is highest in immature sperm from males with abnormal semen values. Also, there is a direct relationship between the ROS generation and the spermatozoa deformation indicator, calculated by dividing the total count of abnormal sperms by the count of sperms evaluated (67).

### The effect of ROS on sperm

**Figure 3:**
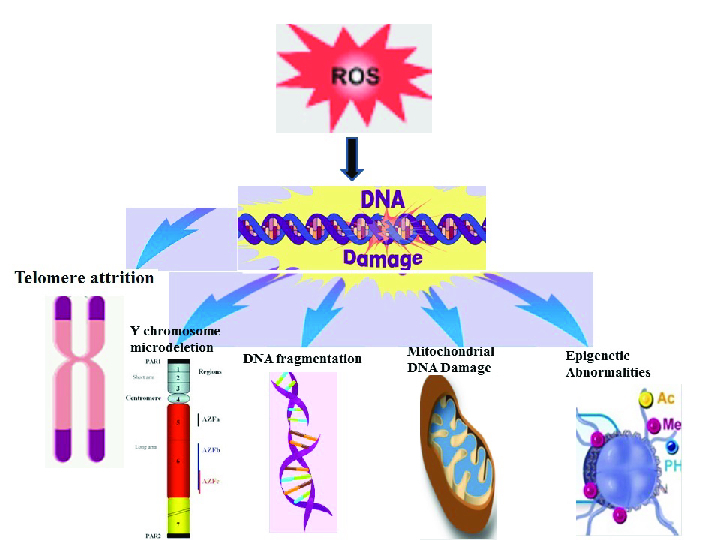
Examples of DNA damage caused by ROS. Excess ROS overwhelms the seminal plasma's neutralizing capacity of antioxidants, triggering OS and, consequently, DNA damage in the nucleus and mitochondria.

Excessive ROS in human semen is severely correlated with male infertility (68). ROS are free radicals derived from oxygen that are required at a low level for capacitation, hyperactivation, motility, and acrosome reaction (69). Nonetheless, excessive ROS level can lead to OS and consequently cause DNA damage, lipid peroxidation, shorting of telomeres, epigenetic variations, Y chromosome microdeletions, and induction of apoptosis (Figure 3) (70, 71). Aitken and Clarkson first observed ROS using the method of chemiluminescence in the human ejaculate (72). Also, high amounts of ROS negatively affect sperm concentration, motility, morphology, and male fertility (67, 73, 74). The sources of endogenous ROS are immature sperm and leukocytes, and there are different external causes (75). Genitourinary infections, varicocele, cigarette smoking, alcohol, recreational drug misuse, ionizing radiation, cell phone usage, stress, excessive exercise, spinal cord damage, parental age, and environmental pollution are all active variables in the OS production (76-78). Sperms are vulnerable to OS because it contains high values of polyunsaturated fatty acids that are sensitive to lipid peroxidation (70). The by-products of lipid oxidization include mutagenic molecules acrolein, malondialdehyde, and 4 hydroxy-nonenal (4-HNE), which indirectly lead to DNA damage (4). 4-HNE and acrolein lead to induction apoptosis and fragmentation of DNA (79). Malondialdehyde has been used in various biochemical studies to track the degree of sperm-related peroxidative damage (79, 80). More of the sperm genome (almost 85%) is linked to central nucleoproteins, which preserves it against free radical attacks (4). Therefore, the plasma membrane is the main purpose of ROS, which triggers a cascade of events that damage the genetic confirmation of sperm (69). In a study conducted by Venkatesh et al., the sperm count, sperm motility percentage, and normal sperm morphology percentage in infertile men reduced significantly in comparison with the control groups. Also, ROS levels in infertile men increased significantly in comparison with the control groups. No significant relation was observed between ROS levels and semen parameters (81).

### Apoptosis as a marker of semen quality

Apoptosis of sperm was considered a potentially useful indicator of male fertility (21). Loss of germ cell, which happens via apoptosis, is a prevailing mechanism during spermatogenesis and is regulated by the expression levels of *p53*, *p21*, *Bcl-2*, *FAS*, and caspases (82). Several studies have reported increased apoptosis rates in samples of poor-quality semen (19). Apoptosis happens during spermatogenesis and has been proven too in ejaculated sperm (83, 84). Studies showed the fragmentation of DNA in ejaculated sperm. OS causes damage to DNA and increased levels of oxidative damage in sperm DNA (85). The measurement of apoptosis may be an index of semen quality (21).

## 4. Conclusion

Apoptosis is one of the well-known mechanisms of quality control in the testis. Nevertheless, increased apoptosis may have adverse effects on sperm production, eventually compromising male fertility. Therefore, controlling the rate of apoptosis for fertility in men is of particular importance. Also, ROS and the consequent OS play a crucial role in apoptosis. ROS have been shown to cause abnormalities in semen analysis and sperm concentration. Therefore, ROS can cause infertility in men.

##  Conflict of Interest

The authors declare that they have no competing interests.
